# A retrospective study on sperm banking: a Uruguayan experience

**DOI:** 10.5935/1518-0557.20180037

**Published:** 2018

**Authors:** Rosina Ordoqui, Natalibeth Barrera, José María Montes, Mariel Canepa, Carla Bonelli, Carolina Surka, Andrea Torrens, Lidia Cantú, Stefan S du Plessis

**Affiliations:** 1 Fertilab Laboratorio de Análisis Clínicos, Laboratorio de Andrología, Montevideo, Uruguay; 2 Medical Physiology, Faculty of Medicine and Health Sciences, Stellenbosch University, Tygerberg, South Africa

**Keywords:** Sperm, banking, cryopreservation, fertility preservation

## Abstract

**Objective:**

The purpose of this study was to investigate the status of homologous sperm banking in Uruguay.

**Methods:**

A retrospective investigation was performed on data collected between 2013 and 2015. Reasons for sperm banking, patient age, pre-freeze and post-thaw semen parameters, and recovery rates were analyzed.

**Results:**

623 samples were cryobanked between 2013 and 2015. Only 324 samples were considered for analysis after selection based on inclusion criteria. In most cases the samples were stored because the patients were undergoing assisted reproductive technology (ART) treatment (n=190; 58,64%) or for oncological reasons (n=113; 34,88%). The median age of bankers was 34 years. In the cancer group, 61.95% (n=70) of the subjects had been diagnosed with testicular cancer. Medians of semen parameters for both groups were above the lower reference limits dictated by the World Health Organization (2010). In fresh samples, a significant difference was observed in progressive motility (47% vs. 56%) between ART and oncological patients. After thawing, total motility (27% vs. 32%), progressive motility (19% vs. 22%), and vitality (48% vs. 56%) differed significantly between ART and oncological bankers.

**Conclusion:**

Semen banking has been performed successfully in Uruguay and outcomes are on par with international standards. Surprisingly, the semen parameters of the cancer group were nearly normal.

## INTRODUCTION

Sperm cryopreservation is a biotechnology widely used in andrology laboratories, particularly those associated with assisted reproduction centers (World Health Organization, 2010). The cryopreservation process involves several steps, beginning with exposure of the tissue or cells to cryoprotectants, followed by subsequent cooling to temperatures below zero and long term storing in liquid nitrogen (LN2) at -196°C in order to preserve viability. Finally, a thawing step is employed and physiological conditions are restored. During this complex procedure cells must maintain their integrity (Agca, 2000). The effectiveness of these techniques depends on the cell type, differing among species and individuals. Therefore, it is vital to comprehend the physiology of the material to be cryopreserved in order to ensure their post-thaw survival and functionality (Woods *et al*., 2004).

Spermatozoa are small cells (22.2±1.2µm^3^) (Curry *et al*., 1996) with extremely condensed nuclei, little cytoplasm, and a very high surface to volume ratio. These characteristics give them the peculiarity of being relatively easy to cryopreserve (Saragusty *et al*., 2011). However, during cryopreservation cells might be damaged for different reasons, including the formation of intracellular crystals, osmotic changes, and mechanical effects associated with the procedure (Centola *et al*., 1992). Despite some variations in results, most authors agree that sperm fertilization capacity might be affected during cryopreservation (Hammond *et al*., 1986; Leeton & Backwell, 1976). Reductions in motility, normal morphology, and sperm viability, as well as structural changes in the acrosome, chromatin defects and reduced mitochondrial activity, have been reported and described by several groups (O'Connell *et al*., 2002; Ozkavukcu *et al*., 2008). The reasons for cryobanking are numerous. The advent of intracytoplasmic sperm injection (ICSI) in high complexity laboratories has significantly increased the dissemination of cryopreservation, particularly in the preservation of testicular and epididymal spermatozoa. Moreover, sperm cryopreservation has also been indicated for patients undergoing vasectomy, vasovasostomy, and recommended for individuals undergoing cancer treatment (Anger *et al.*, 2003).

Sperm freezing techniques have been used in andrology laboratories for many years (Liu *et al*., 2016). Fertilab, the only sperm bank in Uruguay, has amassed more than 28 years of experience with this technology to help preserve male reproductive capacity. Since opening its doors in 1987, Fertilab has banked more than 1528 homologous semen samples. The number of cases has increased through the years, and exponential growth has been recently observed, as 139 samples were banked in 2015 versus 77 in 2012 (81% increase in four years). To our knowledge, there currently is very little data available on the Uruguayan experience with sperm banking. This retrospective study aimed to illustrate the status of homologous sperm cryopreservation in the only sperm bank in Uruguay. Furthermore, the results obtained from our laboratory were discussed in great detail by comparing them to data from various similar studies published in the literature. Finally, our results were used to determine possible shortcomings of our sperm banking system, find solutions to improve the technical aspects of cryopreservation, and offer recommendations to promote a wider adoption of the technology in Uruguay.

## MATERIALS AND METHODS

### Study Population

Our institutional review board approved the study. A total of 623 samples were cryobanked between 2013 and 2015 at Fertilab, including ejaculated semen samples and samples taken from testicular biopsies. The study included only the first ejaculated semen sample banked for each individual patient, on which cryotolerance tests were performed. The included samples were collected prior to the onset of gonadal toxicity and the start of radiation therapy. Samples from patients under 18 years of age were excluded from the study. In the end, 324 samples met the inclusion criteria.

Reasons for sperm banking, patient age, pre-freeze and post-thaw semen parameters, and recovery rates were reviewed. Semen samples were manually analyzed, according to the guidelines prescribed by the laboratory manual on semen analysis published by the World Health Organization (WHO) (World Health Organization, 2010). Concentration, total sperm motility, progressive motility, and vitality (by eosin-nigrosin staining) were recorded before cryopreservation

### Semen cryopreservation

Ejaculates were cryopreserved according to the standard protocol followed at Fertilab. Semen samples were quickly divided into two equal parts and washed in Earle's balanced salt solution (EBSS) without HEPES by centrifugation (300xg, 5 min). The supernatant was removed and EBSS was added to the pellets in a single tube (final volume 0.5mL). Freezing medium (TYB, Irvine Scientific) was added to a final ratio of 1:1. The samples were incubated for 20min at room temperature (RT) and were then loaded into 0.25µL straws. Subsequently the straws were placed in nitrogen vapor for 20 min before being dipped in LN2 and stored at -196°C. After 1min, a straw from each sample was thawed for cryotolerance testing. The frozen aliquots were thawed according to the laboratory's standard protocol. The samples were kept for 3 min in a pre-warmed conical tube at 40°C. Thereafter the content of the straw was expelled into a different conical tube for immediate routine sperm analysis. Post-thaw sperm motility and vitality were assessed. Recovery rates for motility (*i.e*., proportion of pre-freeze sperm that remained with progressive motility immediately after thawing) and recovery rates for viability (*i.e*., proportion of pre-freeze sperm that remained vital and immotile immediately after thawing) were subsequently determined.

### Statistical Analysis

Statistical analysis was performed on software package XLSTAT (Addinsoft, USA). Data normality was assessed through the Shapiro-Wilk test. Since the variables did not follow a normal distribution, the differences between pre-freeze and post-thaw semen parameters were assessed through the Friedman test. Comparisons between groups were made using a non-parametric Kruskal-Wallis test followed by Dunn's multiple comparisons test. Data were expressed in the form median values and interquartile ranges. Differences with *p*-values <0.05 were considered statistically significant. Qualitative data were expressed as proportions and total number of samples under a specific category.

## RESULTS

### Reasons for sperm banking

This retrospective study examined 324 ejaculated samples banked from 2013 to 2015. In most cases the samples were stored because the patients were undergoing assisted reproductive technology (ART) treatment (n=190; 58.64%) or for oncological reasons (n=113; 34.88%). The median age of bankers was 34 years (28-40); as expected, the subpopulation of pre-vasectomy bankers was older and had a median of 44 years (40-45). Bankers in the cancer group were significantly younger than all other banker groups (including ART and pre-vasectomy individuals), with a median age of 28 years (22-33); approximately 75% of the male cancer patients were aged 33 or younger.

In the cancer group, 61.95% (n=70) of the subjects had been diagnosed with testicular cancer, followed by lymphoma (Hodgkin's and non-Hodgkin's lymphomas) (12.39%; n=14), leukemia (3.54%; n=4), thyroid tumors (3.54%; n=4), bone marrow aplasia (2.65%; n=3), prostate tumors (2.65%; n=3), lung tumors (0.88%; n=1), and melanoma (0.88%; n=1). Surprisingly, 3.54% (n=4) of the bankers did not know the type of cancer they had, while another 7.96% (n=9) banked sperm before initiating chemo or radiation therapy without providing information on cancer type. The 190 samples in the ART group were banked as precautionary backup. In this group, 3.16% (n=6) of the men banked sperm because of their possible absence during the time of treatment (regular trips; living in different towns), while the rest of the cohort stored because sperm parameters are known to oscillate or due to potential difficulties having semen samples collected on treatment day.

Table 1 summarizes the seminal parameters of the ART, oncological and total groups before freezing. Significant difference was found only in progressive motility between the ART and cancer groups. No differences were found in concentration, although 25% of the ART bankers had sperm concentrations below 2.5x10^6^/mL and 25% of the cancer patients had concentrations below 7x10^6^/mL. Interestingly, the two groups had semen parameters above the lower reference limits published in the 2010 WHO guidelines (WHO, 2010). Cryptozoospermic specimens (ART n=7; Oncological n=4) were not considered in the calculation of median values.

**Table 1:** Pre-freeze and Post-thaw semen parameters of ART, Oncological and Total Group.

Parameter	ART Treatment n=183	Oncological n=109	Total n=313
**Pre freeze**			
Semen Volume (ml)	3 (2-4)	2.5 (2-3.9)	2.5 (2-4)
Count (x 10^6^/ml)	20 (2.55-48.0)	18 (7-45)	20 (4-50)
Total Motility (%)	59 (42.50-71)	64 (50-72)	62 (47-72)
Progressive Motility (%)	47 (28.50-61.50)*	56 (41-64)	53 (34-64)
Vitality (%)	90 (82-94.50)	92 (87-95)	91 (83-95)
**Post-thaw**			
Total Motility (%)	27 (17-36)*	32 (21-42)	28 (17-38)
Progressive Motility (%)	19 (9-29)*	22 (14-35)	21 (10-31)
Vitality (%)	48 (30.5-61)*	56 (39-67)	49 (32-64)
**Recovery rate (%)**			
Progressive Motility	41.3 (24.3-58)	47.4 (31.9-58.6)	44.4 (25-58.6)
Vitality	56.1 (39-68.9)	61.1 (46.2-73.2)	57.3 (40.8-70.8)

* *p*<0.05 vs. cancer patients by Dunn's multiple comparisons test.

### Semen Parameters before and after cryopreservation

The proportions of spermatozoa showing total and progressive motility, the proportions of live spermatozoa (vitality) before and after cryopreservation, and recovery rates are shown in Table 1. Only pre-freeze progressive motility differed significantly between the ART (47%) and cancer (56%) groups. Post-thaw total and progressive motility and vitality were significantly different between the ART and cancer groups (*p*˂0.05), with specimens from the cancer group consistently displaying higher values. As expected, a significant reduction occurred in all three pre-freeze semen parameters compared to their respective post-thaw values (*p*˂0.05). The calculated recovery rates for progressive motility and vitality did not differ between the three groups. Interestingly, the cancer group had slightly better recovery rates than the ART group.

The samples were subsequently stratified into four groups based on motility and concentration profiles (Normozoospermia, Oligzoospermia, Asthenozoospermia, and Oligoasthenozoospermia) according to the WHO 2010 reference values. The recovery rates based on progressive motility and viability were calculated and compared between groups (Table 2). The recovery rates for progressive motility and vitality were significantly higher for normozoospermic samples versus any of the other three groups. No differences in recovery rates were found between samples having one (oligo- or asthenozoospermia) or two (oligoasthenozoospermia) affected semen parameters before freezing.

**Table 2:** Recovery rates for progressive motility and vitality according to semen quality.

Parameter	Normozoospermia (n=149)	Oligzoospermia (n=153)	Asthenozoospermia (n=77)	Oligoasthenozoospermia (n=55)
**Recovery rate (%)**				
Progressive Motility	49.18 (34.62-60)	33.3 (16.18-54.05)*	36.67 (12.50-60)*	21.43 (0-57.5)*
Vitality	62.5 (47.42-75.26)	48.45 (31.91-67.37)*	47.76 (24.14-62.37)*	40.74 (20.32-58.57)*

* *p*<0.05 vs. Normozoospermia

Oligozoospermic specimens were further sorted into groups based on severity (mild: 10-15x10^6^/mL, moderate: 5-10x10^6^/mL; and severe: less than 5x10^6^/mL) and their sperm parameters before and after cryopreservation were compared and recovery rates calculated accordingly (Table 3). Samples categorized as severely oligozoospermic showed significantly less progressive motility and vitality before freezing and after thawing. Interestingly, the cryosurvival recovery rates of this group were also significantly lower than the rates seen in the groups with mild and moderate oligozoospermia (*p*<0.05).

**Table 3:** Pre-freeze and post-thaw semen parameters of mild, moderate, and severe oligozoospermia samples.

Parameter	Mild	Moderate	Severe
	(10 - 15 x 10^6^/ml) n=22	(5 - 10 x 10^6^/ml) n=39	(˂ 5 x 10^6^/ml) n=92
**Pre-freeze**			
Progressive Motility (%)	55 (41- 65.75)*	47 (33.5- 57.5)*	33 (17- 44)
Vitality (%)	94 (87.25- 95)*	92 (87- 95)*	86 (68.5- 93)
**Post-thaw**			
Progressive Motility (%)	26 (19.25- 31)*	20 (13- 28.5)*	15 (9- 10.17)
Vitality (%)	49 (33.75- 64)*	48 (40 -62)*	32 (17.5 -49.5)
**Recovery rate (%)**			
Progressive Motility	44.4 (37.45- 54.84)*	42.31 (29.35- 64.41)*	22 (10.5- 48.6)
Vitality	53.31 (42.33- 68.29) *	55.91 (47.41- 68.13)*	41.26 (27.27- 64.46)

* *p*<0.05 vs. severe by Dunn's multiple comparisons test.

## DISCUSSION

Over the last few decades, improvements in medical reproductive technology contributed significantly to increase the number of sperm bankers, particularly since the introduction of ICSI. Fertilab established the first human sperm bank in Uruguay in 1987 (Fertilab, 2016). To date, 1397 patients have had sperm samples stored with Fertilab. Although the technique is well established globally and locally, and despite the increase in utilization observed over the last few years in Uruguay, the general population and even some medical professionals are still not familiarized with the technique and its applications. A clear example of the magnitude of this problem is that only 6.4% of the 2038 male individuals aged between 15 and 40 years diagnosed with cancer in the 2007-2011 period registered with the National Cancer Institute of Uruguay (Barrios *et al*., 2014) used cryopreservation to help preserve their fertility. Therefore, finding out more about the Uruguayan cryobank population might help identify gaps and pinpoint specific areas (ART centers, oncological professionals, and urological professionals) where information on sperm cryopreservation is lacking, so as to help highlight the potential benefits of cryobanking.

In our laboratory, the primary group resorting to sperm cryopreservation comprises infertile couples looking to store sperm prior to ART (58.64%). Previous studies have reported similar findings for fresh or frozen sperm used in ICSI cycles (Cayan *et al*., 2001; Kalsi *et al*., 2011). Sperm banking for this reason is usually limited to a few months to help reduce male anxiety and ensure there is a backup sperm sample is available on the day of ART. Some couples undergo ART treatment when the man is not physically present during egg retrieval or at the time the woman is fertile, and in such cases semen cryopreservation might be the only solution (Ping *et al*., 2010).

Cancer patients were the second largest group (34.87%) to seek semen cryopreservation. The survival rates of most cancer types continue to improve with the advancement of treatment options in recent decades (Pacey & Eiser, 2011). Consequently, the number of men of reproductive age suffering from malignant diseases has grown. The preservation of fertility before and after treatment is very important for many of them (Williams, 2010). According to our results, the median age of the subjects storing semen samples prior to cancer treatment is 28 years (33-42), making them significantly younger than the individuals in the ART group (median age of 34 years, ranging between 28-40 years). Similar results have been reported in the literature (Hotaling *et al*., 2016; Tomlinson *et al*., 2015). This clearly demonstrates the desire these patients have to preserve their chances of becoming parents, and the notion that sperm cryopreservation is the only option they have to fulfill their aspirations of parenthood.

The most frequent types of cancer reported in our laboratory were testicular tumor (61.95%) and lymphoma (12.39%). Our results are in agreement with previous findings reported in the study by Depalo *et al*. (2016), in which patients with testicular cancer (seminoma) and Hodgkin's Lymphoma topped the list of oncological cryobank users. A possible explanation for this phenomenon is that the prognosis for these cancer types is better, and therefore patients are more likely to be encouraged by their treating physicians to opt for fertility preservation. The National Cancer Institute in Uruguay reported that 412 patients between the ages of 15 and 44 years were diagnosed with testicular cancer from 2007 to 2011. During the same period, 82 patients with this condition had sperm specimens cryopreserved at Fertilab, thereby accounting for 20% of the population diagnosed with testicular cancer in the nation. Surprisingly, the proportion of patients with other cancer types banking at Fertilab was lower: 12 of 79 (15.2%) individuals diagnosed with Hodgkin's Lymphoma; 10 of 190 (5.3%) subjects diagnosed with Non-Hodgkin's Lymphoma; and four of 117 (3.4%) patients diagnosed with colon cancer. From this picture, it appears that clinicians and patients see testicular cancer as having a stronger correlation with infertility than any of the other cancer types, since a higher proportion of individuals with this disease have sought cryopreservation services. The literature shows an evident association between testicular cancer and impaired fertility in the form of lower sperm concentration levels and fewer live births (Baker *et al*., 2005). However, this perception tends to overlook a population of young men suffering from a variety of malignant diseases and leave them vulnerable, as cancer treatment engenders a roster of complex impacts on fertility. And health professionals often underestimate these impacts.

The third largest category of cancer-related reasons for sperm banking included patients looking to cryopreserve sperm before the start of cancer treatment (chemotherapy or radiation therapy). The exact types of cancer these patients were diagnosed with are unknown, thus illustrating the inexistence of proper communication between oncologists and infertility physicians. It is well known that cancer treatment is frequently aggressive and might produce several side effects, including significant threats to male fertility potential. It is impossible to predict the impact of cancer treatment on fertility, as it might cause transient or permanent infertility (chemotherapy: factors include patient age, drug type and dosage; radiation therapy: factors include the site of irradiation, dosage, and type). For these reasons sperm cryopreservation prior to cancer treatment is highly recommended for patients desiring to have a chance of becoming biological parents in the future (ASRM, 2013). Unfortunately, our data indicated that only a small proportion of cancer patients have sperm specimens cryopreserved. This finding is corroborated by studies carried out in developed nations (UK) (Zapzalka *et al*., 1999). Three factors appear to be connected to this phenomenon: (i) lack of awareness, since oncologists and patients do not comprehend the adverse impact of cancer treatment on germ cells and fertility, which leads oncologists not to refer patients and patients not to seek sperm cryopreservation; (ii) patients focus exclusively on having treatment for their oncological conditions, thereby ignoring future fertility plans and disregarding sperm cryopreservation prior to the start of treatment; and (iii) the discouraging price tag attached to cryobanking and long term storage (Ping *et al*., 2010). These patients in particular should use sperm banks. It is crucial that everyone involved understands that banking implies a chance of future parenthood. The mere fact that oncologists discuss fertility preservation with their patients might indicate that the disease might offer a better prognosis. This might impact patient self-esteem and self-preservation, which in turn might work as psychological protection (Pacey & Eiser, 2011).

Compared to other cell types, spermatozoa are less sensitive to cooling processes due to the high fluidity of their membrane and their low water content (≈50%). However, despite these characteristics, cryopreservation produces deleterious effects on spermatozoa structure and functionality (Oberoi *et al*., 2014), causing decreases in motility, viability, chromatin stability, and membrane integrity, thus inducing morphological alterations (Hammadeh *et al*., 1999).

Loss of sperm motility is one of the parameters commonly reported in the literature (Sharma *et al*., 2015), in addition to used cryoprotective medium, freezing technique, specimen quality, and specimen resistance to freezing and thawing. On average, only 50% of motile sperm survive the freeze-thaw process (World Health Organization, 2010). A loss of 20% in total motility is common for donor samples (Matorras & Hernández, 2007), while sample quality decreases dramatically in patients whose initial semen parameters fall outside normal ranges (Hammadeh *et al*., 1999). Table 1 summarizes the pre-freeze and post-thaw seminal parameters according to three groups (Total, ART and Oncological). The rate of recovery of progressive motile spermatozoa was close to 45% for the total population, which is lower than what has been reported in the literature (Creemers *et al*., 2011; Paras *et al*., 2008). However, when analyzing data using only normal samples according to the 2010 WHO reference values (Table 2), this proportion increases to 49.19% and reaches commonly reported levels. Surprisingly, the semen parameters of the cancer group were nearly normal (Table 1). A variety of studies have reported that cancer adversely affects semen quality (Colpi *et al*., 2004; Howell & Shalet, 2005). However, results are contradictory and similar sperm quality in men with and without cancer before cryopreservation has been described (Rofeim & Gilbert, 2004). It is well known that the parameters of the semen sample before freezing determine, among other things, the success of cryopreservation. It has been observed that in approximately 50% of the individuals with malignant disease semen sample quality is affected even before the onset of chemo or radiation therapy.

It is well known that males contribute to 50% of the infertility cases; male factor is involved when one or more semen parameters are abnormal. Oligozoospermia (<15 million/mL) reportedly occurs in 5% to 18% of subfertile men, while the incidence of specimens with asthenozoospermia (<40% total motility) is seen in 35.5% to 51% of the cases. Abnormal semen parameters often manifest in combination with a condition known as oligoasthenozoospermia, which can be found in up to 21.5% of semen samples of individuals seeking infertility treatment (Acacio *et al*., 2000; Aleisa, 2013). In a study performed in Brazil, a significant reduction in sperm motility was observed after cryopreservation was offered to a group of patients with baseline concentration and motility values below normal levels as established by the WHO in 1999 (Esteves *et al*., 2003). The post-thaw sperm recovery rate was close to 20% and the vitality index reached approximately 52%; these values were in agreement with the values reported in this study in patients with oligoasthenozoospermia (Table 2). The analysis of our data revealed that there were no significant differences (*p*>0.05) in the post-freeze vitality and motility indices between samples with one or two altered baseline semen parameters (oligo- or astheno- vs. oligoasthenozoospermia) (Table 2). In addition, no significant differences were observed in the recovery rates of oligo- or asthenozoospermic samples (*p*>0.05). However, these rates were significantly reduced when compared to normozoospermic samples. One might assume that fresh samples with either subnormal concentration or motility might indicate poorer post-thaw recovery rates regardless of the affected baseline parameter. These results are in agreement with the findings published by Huang *et al*. (1991), in which significant differences were observed in the survival rate of oligozoospermic vs. normozoospermic samples (39.0% vs. 73.3%). These authors also found that the survival rate of oligoasthenozoospermic specimens was different from normal specimens (35% vs. 73.3%), but not from specimens in which only concentration was affected.

It is widely accepted that sperm viability is affected by cryopreservation. As confirmation to this notion, a decrease in viable cells (vitality index = 50%) has been reported in samples from proven fertile and subfertile men (Vieira *et al*., 2012). Interestingly, outcomes appear to improve when rapid cryopreservation protocols are used in comparison to slow freezing methods (64.8% vs. 50.4%) (Vutyavanich *et al*., 2010). Our post-thaw viability recovery results (Normozoospermic = 62.5%; Oligozoospermic = 48.45%) were very much in agreement with these and other reports in the literature.

The capacity to cryopreserve oligozoospermic samples has numerous advantages, including storage of samples from patients with severe oligozoospermia (Schuster *et al*., 2003). The introduction of ICSI has changed the outcome of many couples facing this diagnosis and who previously required donor samples. Findings suggest that it is important to advise men with severe oligozoospermia of the possible decay in sperm parameters, sperm count in particular. This phenomenon might more than likely cause 40% of the oligospermic population to become azoospermic. Therefore, recommendation for sperm cryopreservation when the initial sperm count is lower than 5 million/mL has been suggested (Kolta *et al.,* 2015). However, specimens of this type are challeging to cryopreserve and traditonal methods seem to be ineffective (Di Santo *et al*., 2012). Our results showed significantly lower post-thaw recovery rates for patients with severe oligozoospermia when the concentration was lower than 5x10^6^/mL compared to moderate and mild cases (Table 3). Vitrification is a fairly new and alternative cryopreservation method that has been suggested for cryopreserving samples, including the ones retrieved after testicular and epididymal biopsies. Abdelhafez *et al*. (2009) carried out an exhaustive literature review on several publications related to sperm vitrification (epididymis and testicular samples). The sperm recovery rate varied between 59-100% and publications reporting fertilization data described rates between 18-67%. It has been demonstrated that the use of vitrification does not appear to be advantageous with normozoospermic samples, with values of motility, viability, DNA fragmentation, and post-vitrification morphology remaining similar to values obtained using a fast freezing protocol (Agha-Rahimi *et al*., 2014). Other strategies need to be developed to optimize the cryopreservation of these valuable samples (epidydimal and testicular sperm and for severe oligozoospermia).

Finally, the importance of sperm cryopreservation in andrology laboratories and assisted reproduction centers is indisputable. This technology can be used successfully in high complexity laboratories, inter alia for storage of semen samples from donors, patients with severe male factor infertility, and preservation of fertility in cancer patients. Our retrospective study illustrated the current landscape of sperm cryopreservation in Uruguay, reflecting the lack of discussions between patients and doctors of fertility preservation options before gonadotoxic therapies. It also hinted that counseling must be improved, and that a stronger interdisciplinary network should be built to include oncologists, psychologists, and reproduction specialists. A second important point is to perform more prospective studies to redesign the current cryopreservation protocols for samples with very severe oligozoospermia in order to obtain better recovery rates for this group of patients. Additionally, in order to develop a better understanding about the use of banked samples, future studies must evaluate the successful application of homologous sperm cryopreservation on the reproductive outcome of ART patients in Uruguay. In conclusion, semen banking is performed successfully in Uruguay and in many aspects the outcomes are on par with international standards.
